# Enhancing the Supervision of Community Health Workers With WhatsApp Mobile Messaging: Qualitative Findings From 2 Low-Resource Settings in Kenya

**DOI:** 10.9745/GHSP-D-15-00386

**Published:** 2016-06-20

**Authors:** Jade Vu Henry, Niall Winters, Alice Lakati, Martin Oliver, Anne Geniets, Simon M Mbae, Hannah Wanjiru

**Affiliations:** a UCL Institute of Education, London, United Kingdom; b University of Oxford, Department of Education, Oxford, United Kingdom; c Amref Health Africa, Nairobi, Kenya

## Abstract

An estimated half of all mobile phone users in Kenya use WhatsApp, an instant messaging platform that provides users an affordable way to send and receive text messages, photos, and other media at the one-to-one, one-to-many, many-to-one, or many-to-many levels. A mobile learning intervention aimed at strengthening supervisory support for community health workers (CHWs) in Kibera and Makueni, Kenya, created a WhatsApp group for CHWs and their supervisors to support supervision, professional development, and team building. We analyzed 6 months of WhatsApp chat logs (from August 19, 2014, to March 1, 2015) and conducted interviews with CHWs and their supervisors to understand how they used this instant messaging tool. During the study period, 1,830 posts were made by 41participants. Photos were a key component of the communication among CHWs and their supervisors: 430 (23.4%) of all posts contained photos or other media. Of the remaining 1,400 text-based posts, 87.6% (n = 1,227) related to at least 1 of 3 defined supervision objectives: (1) quality assurance, (2) communication and information, or (3) supportive environment. This supervision took place in the context of posts about the roll out of the new mobile learning intervention and the delivery of routine health care services, as well as team-building efforts and community development. Our preliminary investigation demonstrates that with minimal training, CHWs and their supervisors tailored the multi-way communication features of this mobile instant messaging technology to enact virtual one-to-one, group, and peer-to-peer forms of supervision and support, and they switched channels of communication depending on the supervisory objectives. We encourage additional research on how health workers incorporate mobile technologies into their practices to develop and implement effective supervisory systems that will safeguard patient privacy, strengthen the formal health system, and create innovative forms of community-based, digitally supported professional development for CHWs.

## Abstract (precis)

CHWs used WhatsApp with their supervisors to document their work, spurring healthy competition and team building between CHWs in the 2 pilot sites. While there was considerable variation in the number of times each participant posted messages—from 1 message to 270 messages—in total they posted nearly 2,000 messages over 6 months. 88% of messages corresponded to at least 1 of 3 defined supervisory objectives of (1) creating a social environment, (2) sharing communication and information, or (3) promoting quality of services.

## BACKGROUND

With an estimated worldwide shortage of 4.3 million health workers, the World Health Organization has strongly advocated the widespread training of volunteer community health workers (CHWs) as part of a broader strategy to address this human resource crisis.^1^ Accompanying the current resurgence of interest in CHW programs are calls for innovative and evidence-based strategies to recruit, train, motivate, and retain these health workers.^2^^–^^5^ Robust studies suggest that CHWs are capable of effectively performing basic yet vital health care activities, but the services they deliver are not always of high quality and thus fail to generate anticipated health impacts.^6^^–^^8^ Along with motivational considerations (for example, financial incentives) and factors related to capacity building (such as recruitment and training), a major determinant of effective CHW performance involves the provision of an enabling work environment, including manageable workloads, adequate supplies and equipment, respect from colleagues and the community, and supportive supervision.^9^

Considerable emphasis has been placed on enhancing supervision as a strategy to improve the work environment of CHWs.^8^^,^^10^^,^^11^ While hierarchical models of supervision emphasizing inspection and control were originally promoted to support health workers, more collaborative supervisory strategies are now widely advocated.^9^ These strategies, referred to as facilitative or supportive supervision, are presently viewed as best practices and typically involve “… record reviews, observations, performance monitoring, constructive feedback, provider participation, problem solving, and focused education.”^10^

In the context of current efforts to develop and strengthen programs for CHWs, supervision has been defined as^12^:

*A process of guiding, monitoring, and coaching workers to promote compliance with standards of practice and assure the delivery of quality care services. The supervisory process permits supervisors and supervisees the opportunity to work as a team to meet common goals and objectives.*

Effective CHW supervisory systems are now viewed in terms of 3 overlapping general objectives^12^:

1. **Quality assurance:** continuous monitoring and improvement of CHW performance through measurement, feedback, and learning to ensure activities adhere to policies and procedures
2. **Communication and information:** communicating, gathering, and sharing information related to CHW activities, health guidelines, and planned events
3. **Supportive environment:** coaching, problem solving, team building, and other activities that provide CHWs with emotional support

CHW supervisory systems have 3 objectives: quality assurance, communication and information, and supportive environment.

Supervision remains among the weakest aspects of many CHW programs.^8^ Studies suggest that supervision of CHWs suffers from “… low coverage; low administrative focus; is irregular, unsupportive, and demotivating; and lacks adequate training for supervisors and problem solving or feedback mechanisms for providers.”^10^ Reported barriers to delivering effective supervision to CHWs include travel expense and logistics for face-to-face meetings, lack of appropriate supervisory tools, inadequate understanding of the roles of CHWs, and the perception that supervision is not a priority area.^12^^–^^14^ Supervisors frequently lack the training and resources to provide a supportive environment for CHWs and their oversight has remained bureaucratic and punitive.^10^ Supervisory systems have been found to be poorly designed, underfunded, and left to the discretion of busy facility staff who fail to understand the role of CHWs.^8^ Enhancing the implementation of supportive supervision that is appropriate for CHWs thus remains a critical step toward extending the reach of the health care system to where needs are the greatest.^15^

Widespread use of mobile phones in low-income countries has created momentum to use these devices to strengthen supervisory systems for CHWs,^12^^,^^15^^,^^16^ who often work in households in the community beyond the confines of a health facility.^17^ Mobile devices may enable supervisors to overcome resource constraints and geographical distances to monitor CHW activity in real time, provide remote guidance, deliver timely feedback, or send automated motivational messages or reminders.^18^ In spite of this widely recognized potential among policy makers, practitioners, and researchers, only a few studies demonstrate the use of mobile devices to support the supervision of CHWs.^19^

Mobile devices may enable supervisors to overcome resource and geographical constraints to monitoring CHW activity.

In this article, we build on existing research by describing how a group of Kenyan CHWs and their supervisors used the WhatsApp mobile messaging platform for supervision and professional development over a 6-month period. The objectives of our exploratory study were to: (1) document use of the WhatsApp technology to support supervision for CHWs; (2) identify what CHWs and their supervisors used WhatsApp to discuss; and (3) explore how this use relates to current policy guidance on supervision in CHW programs generally.^12^

## PROJECT CONTEXT

Data for this study come from a larger mobile learning intervention—mCHW (http://www.mhealthpartners.org/projects/mchw/)—to strengthen supervisory support for CHWs in Kenya (or “CHVs”—community health volunteers—as they are known in Kenya). The mCHW intervention was designed by education researchers at Oxford University and UCL Institute of Education, in partnership with AMREF Health Africa. By drawing from the disciplinary fields of lifelong learning, computer-supported collaborative work, and mobile learning, mCHW aimed to build on AMREF Health Africa’s 3 decades of experience in delivering training and e-learning programs for health workers in sub-Saharan Africa.^26^^–^^29^ The project involved 3 cycles of design, development, implementation, and evaluation of a mobile learning prototype for CHWs and their supervisors (called the REFER App) that related to child development milestones. The WhatsApp learning group was not specified in the original design of the mobile learning intervention. Rather, it was separate from the prototype and was established at the beginning of the first design cycle, in response to the enthusiasm of project participants who worked in different sites and wished to extend the productive interactions that had taken place during prior face-to-face mCHW workshops.

### Site Selection

The mCHW project purposively selected 2 study sites in Kenya. One site was in Makueni, a semi-arid rural county with an estimated 61% of its population living below the poverty level.^30^ The second site was based in Kibera, one of the largest urban informal housing settlements in sub-Saharan Africa.^31^

### Choice of the Technology Platform

Our choice of WhatsApp reflected existing patterns of technology use in Kenya, where an estimated 49% of mobile phone users use WhatsApp as their preferred mobile messaging tool.^20^ This cross-platform application for basic, feature, and smart phones requires a mobile Internet connection to operate, allowing users to send and receive text messages, photos, videos, and audio recordings. Launched in 2009, it has reached 900 million active users around the world in less than 6 years.^21^ Popular media attributes the global popularity of WhatsApp to its ease of use and affordability.^22^^,^^23^ For an annual subscription fee of US$0.99, users communicate with individuals or groups without incurring additional charges other than the cost of data, with no upper limit on the number or length of messages sent or received. Such mobile instant messaging tools are often referred to as “Over the Top” (OTT) applications because they support communication between users irrespective of the cellular network or mobile device being used.^24^

Nearly half of mobile phone users in Kenya use WhatsApp for mobile messaging.

Short message service (SMS)-based communication typically consists of 2-way interaction between a single user and a single receiver, or it requires specialist software and extra costs to enable broadcast services. In contrast, OTT instant messaging tools such as WhatsApp are designed for less costly, multi-way communication, with functionalities that readily support 1- and 2-way interaction at the one-to-one, one-to-many, many-to-one, or many-to-many levels. A recent literature review suggests that most mobile health projects for CHWs employ SMS-based strategies involving 1- or 2-way interaction, whereas few projects have adopted multi-way communication strategies to promote health priorities.^25^ Our aim was to understand the dynamics of these multi-way communication exchanges and the nature of supportive supervision that could be realized in such an environment.

### Study Participants

We created a WhatsApp group for CHWs, their supervisors (known as community health extension workers, or CHEWs), and the project team members. Each mCHW participant was enrolled as a member of this WhatsApp learning group upon assignment of a project mobile phone and completion of a training session on the use of a (separate) mobile application designed by the project to assess childhood development milestones.

We created a WhatsApp group for CHWs and their supervisors to support communication and team building.

Access to the closed WhatsApp learning group was strictly moderated by the mCHW study manager. Otherwise, communication was informally monitored by the supervisors. There was no fixed schedule for posting new content and all members of the group were encouraged to send messages to the group at any time. It was envisioned that this group would serve as a collaborative learning forum for: (1) team building between Makueni and Kibera participants; (2) additional communication with supervisors; and (3) troubleshooting and sharing experiences related to use of REFER App, the mCHW mobile learning application.

## METHODS

This exploratory descriptive study was conducted during the design phases of mCHW, as part of refining the mobile prototype to train and supervise CHWs. The analysis strategy adopted a qualitative research approach and reflects the exploratory and participatory objectives of qualitative inquiry as practiced in naturalistic, nonexperimental settings.^32^^,^^33^ Our analysis relied on 2 sources of qualitative data: (1) text messages sent in the WhatsApp learning forum, and (2) transcripts of interviews with CHWs and their supervisors.

### Text Messages Sent in the WhatsApp Learning Forum

#### Data Collection and Management

We analyzed the first 6 months of chat logs from the WhatsApp learning group. Electronic data from August 19, 2014, to March 1, 2015, were downloaded from the WhatsApp platform and exported into a Microsoft Access database. We linked each post to a master file containing variables for job titles and the work locales of chat participants and then removed individual identifiers from the data set.

#### Data Analysis

One researcher used the NVivo data software for qualitative research to analyze the text of the WhatsApp posts. First, the researcher identified themes that emerged by analyzing chat conversations using an inductive, qualitative content analysis process.^34^ Three iterations of coding took place as part of this inductive stage of analysis. The first round involved *open coding*, whereby each post was read by the researcher and iteratively assigned 1 or more codes, based on the subject of the message, both on its own and in relation to the posts immediately preceding and following it. This is not intended to suggest that every post carries equal weight with respect to the importance or volume of each message. Some posts contained single words or icons while others were lengthier. Individual posts in their aggregate corresponded to larger episodes and more complex communication exchanges. Our approach to coding and summarizing the content necessarily masks these important nuances of technology use and communication in order to foreground participants’ purposes in using WhatsApp, a limitation of which we are aware. The second round involved *categorization*, combining redundant and related codes into broader, higher-order groupings. During the third round of inductive coding, the number of codes/categories was further reduced to formulate general themes, as part of the procedure known as *abstraction.*

This inductive analysis was accompanied by a deductive approach to explore how the use of WhatsApp corresponded to current conceptual thinking about supervision in CHW programs. We drew specifically on work by Crigler, Gergen, and Perry,^12^ who have investigated how supervision of CHWs is related to health systems strengthening. They propose that supervision systems specifically designed for CHWs must have the 3 main objectives mentioned previously: (1) quality assurance; (2) communication and information, and (3) a supportive environment.^12^ Based on the codes that were assigned during the inductive analysis, each WhatsApp post was therefore allocated to 1 or more groups corresponding to these 3 stated supervisory objectives. Frequency statistics were then calculated for codes and categories using NVivo. Summary tables and graphics were produced using Microsoft Excel.

### Transcripts of Interviews With CHWs and Their Supervisors

#### Data Collection and Management

Semi-structured interview questions were used to explore participants’ views on their use of WhatsApp for facilitating supportive supervision. Interviews were undertaken in the local communities, usually at the local health center or dispensary. Fifteen semi-structured interviews (with 4 supervisors and 11 CHWs) were carried out by a second researcher in March 2015. This was followed by 19 additional interviews (with 4 supervisors, 11 CHWs, 2 community leaders, and 2 public health officers) in June 2015.

Questions on the use of WhatsApp were asked as part of a longer interview structure. The entire interviews lasted between 30 and 35 minutes, with discussion of WhatsApp use taking 5 to 10 minutes of this time. We asked about WhatsApp use directly during the interview (e.g., Have you used or do you use WhatsApp to communicate with your CHVs/CHEWs?), but in other cases health workers volunteered their views on WhatsApp without prompting, as part of discussing their role in mCHW more generally.

### Data Analysis

These interviews were transcribed and coded by the second researcher for instances of “WhatsApp,” and these events were then process-coded to detail how this mobile messaging tool was used for supportive supervision. This coding took place using NVivo, as part of a more comprehensive coding scheme to analyze broader CHW roles and practices. The subset of interview data on WhatsApp usage is presented here to provide additional insight into the findings from the content analysis of the text messages.

### Research Ethics

This study was reviewed by 2 institutional review boards and adhered to the respective codes of ethics adopted by the British Educational Research Association and AMREF Health Africa. Both sets of ethical guidelines require informed consent before data are collected, guarantees of confidentiality and anonymity for participants, as well as the right of participants to withdraw and have their data removed. The ethical protocol, including briefing sheets and informed consent forms, received approval from the lead institution’s ethical review board and from AMREF Health Africa.

Care was taken to ensure that all participants understood that they were acting as volunteers and were not obligated to participate in the project. The WhatsApp m-learning group was a closed chat group, and the project study manager moderated strict access. All WhatsApp users were instructed to obtain verbal consent before posting photos of individuals. Prior to conducting the analysis for this paper, personal identifiers were removed from chat logs, and results presented cannot be attributed to any individual participant.

## FINDINGS

### Description of WhatsApp Users and Number of Messages Posted

During the first 6 months of deployment, a total of 41 individuals joined the WhatsApp learning group and posted at least 1 message. The group was used extensively, with a total of 1,830 posts made during the 6-month study period.

41 members of the WhatsApp group posted 1,830 messages over 6 months.

**FIGURE 1:**
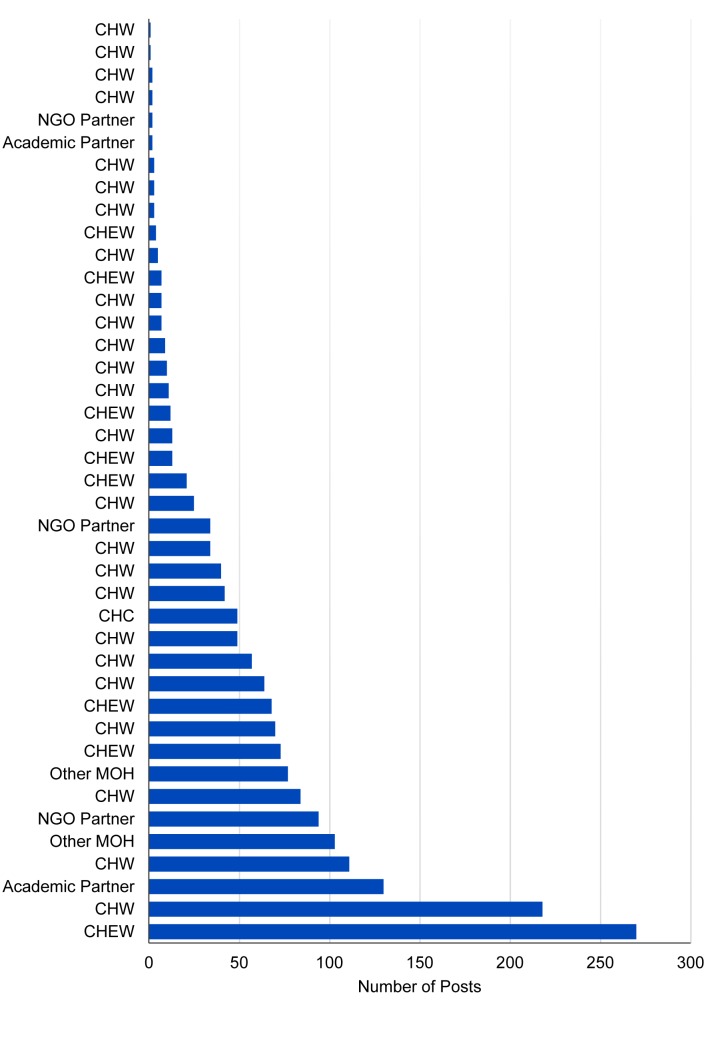
Number of Messages Posted by Each WhatsApp Learning Group Participant, Kibera and Makueni, Kenya, August 19, 2014 – March 1, 2015 (N=1,830 Messages) Abbreviations: CHC, Community Health Committee; CHEW, community health extension worker; CHW, community health worker; MOH, Ministry of Health.

Of the 41 participants, 61% (n = 25) were CHWs, while 20% (n = 8) were supervisors and 5% (n = 2) were from other Ministry of Health entities. The remainder were from the NGO partner organization (n = 3, or 7%), the academic partner institutions (n = 2, or 5%), and the Community Health Committee (CHC) (n = 1, or 2.4%). Participants who were not academic and NGO partners were almost evenly divided between the study sites in Kibera (n = 17) and Makueni (n = 19). There was considerable variation in the number of times participants posted messages, with 1 individual sending as many as 270 messages, while others posted only once during the 6-month study period (Figure 1).

The number of times participants posted messages to WhatsApp ranged from 1 to 270.

**FIGURE 2:**
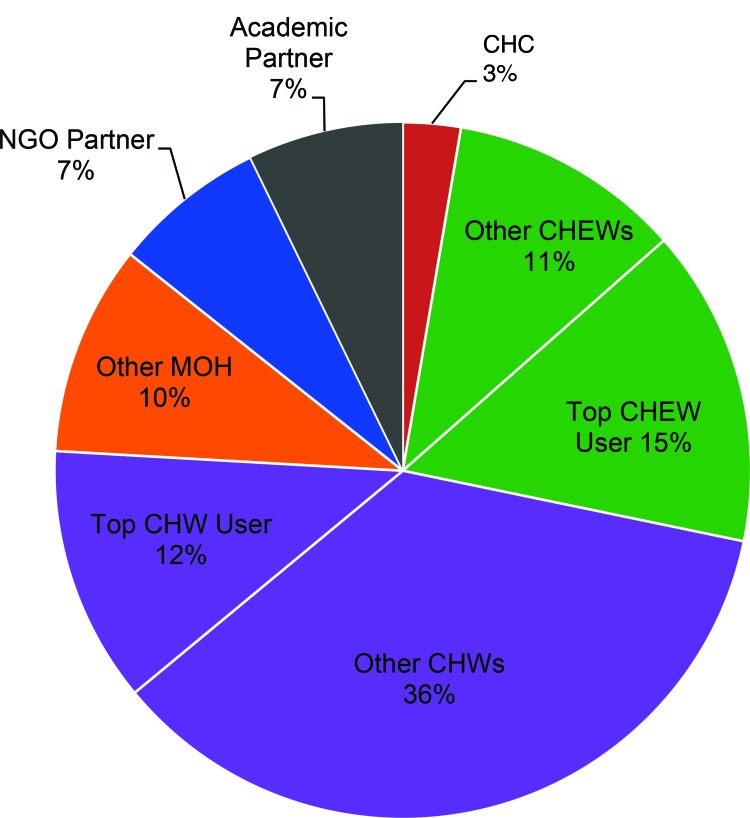
Percentage of All WhatsApp Learning Group Messages Posted by Job Type of Senders, Kibera and Makueni, Kenya, August 19, 2014 – March 1, 2015 (N=1,830 Messages) Abbreviations: CHC, Community Health Committee; CHEW, community health extension worker; CHW, community health worker; MOH, Ministry of Health.

CHWs posted 48% (n = 872) of all messages, with 1 CHW alone posting 12% (n = 218) of these messages (Figure 2). One supervisor posted 15% (n = 270) of all messages, with the remaining supervisors posting 11% (n = 198) of messages, for a combined total of 26% (n = 468). The NGO and academic partners each posted roughly 7% of the total number of messages each (n = 130 and n = 132, respectively), while other MOH representatives at the district and local level posted 10% (n = 179) of messages and the local CHC leader 3% (n = 49) of messages.

Interviews suggested that in general the use of WhatsApp was viewed very positively and was taken up very easily by participants. For example, 1 supervisor remarked:

*WhatsApp has been the best thing ever and I wouldn’t have guessed the community health volunteers would adapt WhatsApp the way they did.*

### Relation of WhatsApp Posts to Supervision Objectives

Using inductive analysis, we grouped the 1,830 total posts into 36 categories (Table 1). After excluding posts of photos, icons, video, and audio (n = 430), we then conducted deductive data analysis to explore the extent to which the remaining 34 types of coded posts could be grouped into the 3 stated objectives of CHW supervision: (1) quality assurance, (2) communication and information, and (3) supportive environment. At least 19 of the 34 categories of codes that we generated during the inductive analysis phase described practices that related to the overall objectives of supervision, with 1,227 of 1,400 (87.6%) coded posts assigned to at least 1 of these supervision objectives (Table 2). Categories created to characterize the substantive areas of the posts, such as “disability” and “malnutrition,” were not included in this analysis. Supervision-related posts were most commonly related to the objective of creating a supportive environment (64.7%), followed by communication and information (33.4%) and quality assurance (19.0%).

88% of WhatsApp posts corresponded to at least 1 of 3 supervision objectives.

**TABLE 1:** Coding Scheme to Describe Messages Posted by WhatsApp Learning Group Participants, Kibera and Makueni, Kenya, August 19, 2014 – March 1, 2015– (N = 1,830 Posts, Codes Listed in Alphabetical Order)

Code	Description	No. of Posts^a^	Code	Description	No. of Posts^a^
**Chain letter**	Generic message forwarded to members	12	**Mobile app**	References to child developmental milestones or the project app	46
**Clarifications**	Corrections to a prior post	11	**Mobile phone**	Operational aspects of using mobile phones	113
**Community development**	Community mobilization efforts in Kibera or Makueni	35	**Moral support**	Condolences and encouragement in response to challenges or hardships	133
**Disability**	Documentation of disabled child’s developmental milestones	111	**Mutual learning**	Informal learning, peer learning, and knowledge exchange	108
**Encouragement and praise**	Encouragement and praise in response to a prior post	281	**Other health initiatives**	References to health programs other than childhood disability, malnutrition, or water and sanitation	110
**Evidence**	Documentation of CHW practice	14	**Other media**	Posts containing only icons, videos, or audio and exclusive of text	19
**Follow-up**	Responses to a prior post or event	120	**Photos**	Posts of photos	411
**Fundraising and donations**	Collection or distribution of money or objects	16	**Photo captions**	Posts containing descriptions of photos	183
**Greetings**	Hellos, welcome messages, and holiday wishes	215	**Referral**	Referrals of patients to CHEW or health facility	79
**Health education**	CHW efforts to educate community	31	**Religion**	Bible verses, blessings, and other references to faith	108
**Household visits**	Encounters during CHW household visits	51	**Reporting**	CHW descriptions of patient encounters or community work	100
**Inspiration**	Words to motivate forum participants	48	**Reprimand**	Posts that discipline or challenge other WhatsApp participants	16
**Job offers and professional development**	Employment announcements and outside training	22	**Requesting information**	Solicitation of additional information from others	83
**Kibera-Makueni exchange**	Communication between Kibera & Makueni participants	137	**Security**	References to community violence, fires, or other disturbances	4
**Kenya**	Nation building	8	**Service**	Statements about community service	6
**Logistics and planning**	Time, date, location, and agenda of meetings and other events	186	**Thanks**	Expressions of gratitude	304
**Malnutrition**	Descriptions of work with malnourished children	16	**Training**	Formal learning opportunities	122
**mLearn project**	References to the mLearn project and its site visits, conferences and training activities	297	**Water and sanitation**	Initiatives to promote clean water and hygiene	42

a More than 1 code may have been assigned to any 1 post, so percentages will not total to 100%.

**TABLE 2:** Categories and Frequency of Text Messages Posted to the WhatsApp Learning Group by Supervision Objective, Kibera and Makueni, Kenya, August 19, 2014 – March 1, 2015

Supervision Objective	Categories of Posts	No. of Posts	% of All Posts (N = 1,400)^a^
Supportive environment	Encouragement and praise	906	64.7%
	Greetings		
	Inspiration		
	Kibera-Makueni exchange		
	Moral support		
	Mutual learning		
	Religion		
	Thanks		
Communication and information	Evidence	467	33.4%
	Job offers and professional development		
	Logistics and planning		
	Reporting		
	Requesting information		
	Training		
Quality assurance	Follow-up	266	19.0%
	Health education		
	Household visits		
	Referral		
	Reprimand		
Any supervision objective	Any of the above codes	1,227^b^	87.6%^b^

a Denominator includes all posts except those containing photos or other media.

b More than 1 code may be assigned to any 1 post, so percentages will not total to 100%.

#### Supervision for a Supportive Environment

Of the 1,400 text-based posts, almost two-thirds (n = 906) corresponded to the provision of a supportive environment—that is, providing CHWs with emotional and motivational guidance, mentorship, and assistance with problem solving. Interviews with participants suggested that the WhatsApp group was well-suited to promote a supportive environment for health workers:

*It has helped me do my supportive supervision a little bit for the community health volunteers, more so for the ones [community health volunteers] who share what they have done or what have they done extra.*

Most of the WhatsApp posts corresponded to the supervision objective of providing a supportive environment.

Within this context, participants confirmed that they viewed the WhatsApp environment as an opportunity for mutual learning:

*The person I am chatting with educates me, and also I educate her or him so it helps me. You know, some send photos and explain about them; therefore, I learn.*

These participants also highlighted the importance of being able “to put different worlds together” to promote learning and motivation:

There’s the exchange of ideas, and people get to learn that these people are working on this project and there’s kind of a competition … which is very healthy. So we have the Makueni, [and] we have Kibera. They [Makueni health workers] post this article [about] what they have visited, and Kibera [workers] also feel they don’t want to be left behind, [so] they do the same. Makueni, the same. And this is very healthy.

#### Supervision for Communication and Information

Supervisors are expected to keep CHWs informed about new guidelines and planned events, as well as to gather service statistics to document the work of these health care providers. Posts related to communication and information, which relates to these data collection efforts and educational activities, accounted for 467 of the 1,400 messages (33.4%). In particular, we found the forum was useful for communicating information related to logistics and planning as well as training. One supervisor explains:

*I think it has been a platform for us to communicate, and actually I can say it has reduced some costs when it comes to communicating with the community health volunteers because if I know people are in the [WhatsApp] group, I don’t have to SMS everyone because the SMSs have a cost.*

With respect to information sharing, WhatsApp was particularly useful during emergency outbreaks. One supervisor describes her experience during a cholera outbreak:

*So the communication is good between us [the supervisors] and the community health workers. We really communicate and in case there is any problem, like when there was this outbreak of cholera, we really shared a lot. You give the information you know, they [the CHWs] give you what they know, and [you] can advise “do this, do this, concentrate on this area,” so that that thing [cholera] can end. This [is] through the WhatsApp.*

The WhatsApp group was particularly useful to share information and obtain guidance during emergency outbreaks.

#### Supervision for Quality Assurance

Quality assurance supervisory activities relate to the “adherence to norms and guidelines and the provision of adequate supplies.” Because a supervisor is often the only regular contact that the CHW has with the formal health system, there is an expectation that the supervisor will make sure that a CHW understands his/her tasks and can perform them to an acceptable standard.^12^ There were fewer messages posted to the WhatsApp learning group to promote quality assurance, comprising 19.0% (n = 266) of 1,400 messages. The supervisors felt these posts helped them to be more aware of what CHWs were doing—and not doing—in the community:

*[WhatsApp] has made me learn a thing or two, it has made me get to know characters as far as community health volunteers are concerned, and how they communicate, and [has made me get to know] the other sides of community health volunteers. And I can even gauge performance when it comes to community health volunteers.*

The WhatsApp platform has proven particularly useful in allowing supervisors to gain a better understanding of the situation on the ground, as well to give feedback to CHWs, as part of an overall supervision process:

*The key role of giving feedback [to CHWs] is to know how we are progressing in doing referrals and doing monitoring. What I know [is] this project is targeting the under-6 who are disabled, so we are able through this feedback [cycle] to know how many children are affected and how many are not affected, and if the children are affected, we are able to make a timely intervention.*

In addition, the interview accounts suggested that interaction related to supervision for the quality of services may have taken place via communication channels other than the WhatsApp learning forum that was established by mCHW members.

Participants from both study sites described the creation of additional WhatsApp groups corresponding to different sets of users, including 1 forum exclusively for public health officers:

*In case you are stuck, you don’t know what to do here, you don’t know what to do anyway, you just communicate immediately [with other public health officers] and you will get the information immediately. So it has really made our work to be easy. … We have our own [WhatsApp group] icon here, then we can even communicate with other colleagues [apart from the mCHW learning group].*

Another supervisor reported:

*We have several [WhatsApp groups] for different projects. We have even one for [health care provider] staff here where we are [in the facility], [and] we have one for other [purposes] like hypertension.*

Beyond the learning group, supervisors also revealed that they regularly used voice calls and WhatsApp in general to communicate one-on-one with individual CHWs to understand more about the services provided to households.

### Supervision in the Context of CHWs’ Everyday Practices

The 36 categories of posts that were generated during the first 2 rounds of inductive coding (Table 1) are revisited here to provide additional contextual insight into the supervisory interactions that took place between WhatsApp participants. Overall, these categories of codes illustrate how interactions were courteous, encouraging, and linked to the practices of CHWs as health cadres and community leaders. As shown in Table 3, 26 of the 36 categories of posts can be grouped to reveal 3 broad themes: posts about the mCHW learning intervention, posts about other CHW practices unrelated to the mCHW learning intervention, and posts for team building and community development.

**TABLE 3:** Categories and Frequency of Messages Posted to the WhatsApp Learning Group by Broad Theme Related to CHWs’ Everyday Practices, Kibera and Makueni, Kenya, August 19, 2014 – March 1, 2015

Theme	Categories of Posts	No. of Posts With at Least 1 Code in Category	% of Posts in Category (N = 1,400)^a^
Posts about mCHW learning intervention	Disability	435	31.1%
	mLearn project		
	Mobile app		
Posts about other CHW practices unrelated to the mCHW learning intervention	Follow-up	445	31.8%
	Health education		
	Household visits		
	Malnutrition		
	Other health initiatives		
	Referral		
	Reporting		
	Water and sanitation		
	Training		
Posts for team building and community development	Community development	819	58.5%
	Fundraising and donations		
	Greetings		
	Inspiration		
	Inspiration Job offers and professional development		
	Kibera-Makueni exchange		
	Kenya		
	Moral support		
	Mutual learning		
	Religion		
	Service		
	Security		
	Thanks		

a More than 1 code may be assigned to any 1 post, so percentages will not total to 100%.

#### Posts About the mCHW Learning Intervention

Of 1,400 text posts, 31.1% contained messages related to roll out of the mCHW intervention. This was one of the primary motivations for setting up the WhatsApp group. A CHW from Makueni posted:

Hi kibra team sometimes u may think we are quiet but u experienced our big challenge is on charging phones, so we realy [sic] request [researcher’s name removed to maintain confidentiality] to put more effort on those “sollarchagers” plz [sic]

Participants sent messages related to passwords and airtime for the project and sent photos and feedback on mCHW training sessions. CHWs also used the WhatsApp group to document actual household visits involving use of the mCHW application to assess developmental milestones of children with disabilities:

*[Child’s name removed to maintain confidentiality] nine months old. she tends to put all what she holds to her mouth. she always scores a pass in her milestones*

CHWs shared photos to the WhatsApp group that captured their use of the mCHW mobile application, along with motivational messages of congratulations and encouragement to keep up the good work.

#### Posts About Other CHW Practices Unrelated to the mCHW Intervention

The WhatsApp posts during this study period were not only about the mCHW learning intervention; there were 445 of 1,400 (31.8%) posts that provided feedback related to other ongoing CHW practices. This included administrative and clinical guidance on how to follow-up with or investigate related conditions, such as malnutrition, experienced by children:

*Thank you [name removed to maintain confidentiality] for posting this information and for the support you have given so far to improve the nutritional status of the baby. I would suggest you refer the Baby to Kikuyu Eye Hospital. I [will] call the team members to come up with more suggestions…*

Participants also used the WhatsApp group to communicate about their ongoing work in water and sanitation, HIV prevention, maternal and child health, and other health initiatives:

*Hi all let's continue educating our community more on proper hygiene and wearing gloves when handling any body fluids rember [sic] to wash your hands with ranning [sic] clean water and soap. wish u all good healthy times.*

Their posts also documented their health education work during community meetings such as “action days” and “dialogue days,” their household visits to care for the elderly, and their presentations at elementary schools.

#### Posts for Team Building and Community Development

Although a substantial number of the posts were devoted to health-related matters, the contributions of the participants to the WhatsApp platform also served to enact an informal and supportive community of learners. Of 1,400 posts, 58.5% related to what we categorized as team building and community development. For example, there were multiple exchanges between the 2 different study sites. Many posts contained enthusiastic announcements related to other training opportunities and job opportunities, as well as reminders about upcoming meetings and workshops. Words of welcome, along with messages of encouragement, praise, and thanks, were commonly posted in response to individual posts to the forum. There were the occasional chain letters, and a multitude and variety of emoticons and small images corresponding to applause, approval, and the various holiday seasons were embedded into the text of many postings.

With 59% of posts relating to team building and community development, the WhatsApp group served as an informal community of learners.

There was also a more sober side to the community-building efforts. The CHWs and their supervisors often posted messages related to community service, a vision for the nation of Kenya, religious prayers, and other allegories intended to inspire and motivate the group. These messages were usually sent in response to posts about the fires, rioting, and personal tragedies that occurred multiple times during the course of the short study period. In those situations of hardship, group participants used the WhatsApp platform to coordinate the logistics of delivering financial and moral support to those other members of their community.

### Use of Photos in WhatsApp Communication and Supervision

Photos were a key component and often the basis of the communication that took place among CHWs and their supervisors: of the total 1,830 posts made during the study period, 23.5% (n = 430) contained photos or other media and an additional 10.0% (n = 183) contained comments or captions related to those photos. Supervisors posted photos of supervisory visits, meetings, and training sessions that had taken place that day, followed by words of praise, motivation, and appreciation. CHWs posted photos to document the quality of services they delivered, with posted messages often referring to the photos as “evidence” of CHW work practices in the community:

*It is the evidence of whatever we do in the community. It has been the best evidence. If I assess a child, I take a photo, or if I have attended any sick person in the community and I post on WhatsApp. … If we [in Kibera] don’t post, they will say in Makueni we don’t work, we don’t see their work.*

Photos posted to WhatsApp often formed the basis of communication between CHWs and their supervisors.

CHWs posted photos of their practice, followed by captions describing the content of the photos. Supervisors followed up with guidance, encouragement, and/or thanks while fellow CHWs added words of praise and encouragement. One supervisor explained:

*So it has really been useful to give that drive to the community health volunteers to perform tasks. The other issue is that like right now every community health volunteer takes a photo when they go to a household, then they post there, then they explain to us what they are doing there.*

A more thorough understanding of what these photos depict, why they are preferred over text-based forms of reporting, and how they are used by groups would be useful as part of strengthening current forms of public health reporting and health information systems.

## CONCLUSION

This study attempted to provide a snapshot of the content that was posted in a WhatsApp group by a cohort of CHWs and their supervisors during a 6-month period. At this preliminary stage, the grouping of the 36 categories of posts into broad themes associated with supervision (Table 2) and CHW practices (Table 3) is intended to provide insight into the kinds of messages that are sent by participants, rather than to develop formal typologies. We recognize that there may be alternative ways to assign the categories into those larger groupings. Furthermore, this analysis does not assess or predict the likelihood or intensity with which CHWs and their supervisors will use an instant messaging platform. Our findings show that there was considerable variation in the number of posts contributed by each of the 41 participants. This could be due to the staggered dates that participants joined the group, which corresponded to the operational roll-out of the mCHW learning intervention. Alternatively, there may be personal preferences or power dynamics related to factors such as gender, age, or position in the community that influence the extent to which CHWs and their supervisors will engage with the WhatsApp group.

Findings from other studies have suggested that providers use multi-way communication channels for education and practice in a range of health settings.^35^^,^^36^ Our content analysis of the messages that were actually posted by CHWs and their supervisors demonstrates how they employed mobile instant messaging technology. They used this tool to exchange information and create content that corresponded to their roles as both health workers and community leaders. These interactions also correspond to stated objectives of supervisory systems for CHWs—that of quality assurance, communication and information, and creating a supportive environment. While there is general consensus about these 3 broad objectives, there is less clarity about whether a single individual should carry out all of such functions.^37^ It has been suggested that new approaches to CHW supervision should allocate the various functions of supervision to different parties while making use of mobile technology.^12^

Our preliminary investigation demonstrates that with minimal training, CHWs and their supervisors tailored the multi-way communication features of this mobile instant messaging technology as part of enacting virtual one-to-one, group, and peer-to-peer forms of supervision, and they switched channels of communication depending on the supervisory objectives. We encourage additional research on how CHWs and their supervisors incorporate WhatsApp and other mobile technologies into their practices to support the development and implementation of effective supervisory systems that will safeguard patient privacy, strengthen the formal health system, and create innovative forms of community-based, digitally supported professional development for CHWs.

With minimal training, CHWs and their supervisors tailored WhatsApp to enable virtual one-to-one, group, and peer-to-peer supervision.

## Notes

**Peer Reviewed**

**First Published Online:** 2016 May 26
